# Wild Mesocarnivores as Reservoirs of Endoparasites Causing Important Zoonoses and Emerging Bridging Infections across Europe

**DOI:** 10.3390/pathogens12020178

**Published:** 2023-01-23

**Authors:** Fabrizia Veronesi, Georgiana Deak, Anastasia Diakou

**Affiliations:** 1 Parasitology Laboratory of the University Teaching Hospital, Department of Veterinary Medicine, University of Perugia, 06124 Perugia, Italy; 2 Department of Parasitology and Parasitic Diseases, Faculty of Veterinary Medicine, University of Agricultural Sciences and Veterinary Medicine of Cluj-Napoca, 400372 Cluj-Napoca, Romania; 3 Laboratory of Parasitology and Parasitic Diseases, School of Veterinary Medicine, Faculty of Health Sciences, Aristotle University of Thessaloniki, 54124 Thessaloniki, Greece

**Keywords:** wildlife, mid-sized carnivores, parasites, parasitic zoonoses, bridging parasitic infections, helminths, protozoa, vector-borne parasites

## Abstract

Mesocarnivores are small- or mid-sized carnivore species that display a variety of ecologies and behaviours. In Europe, wild mesocarnivores are represented by the red fox (*Vulpes vulpes*), the golden jackal (*Canis aureus*), the European wildcat (*Felis silvestris*), the Mustelidae of the genera *Meles*, *Martes*, *Mustela*, *Lutra*, the invasive species of raccoon dog (*Nyctereutes procyonoides*), raccoons (*Procyon lotor*), and American mink (*Neogale vison*). These abundant animals thrive in various habitats and often develop their activity close to human settlements. Thus, they may play an important role in the introduction, maintenance, and transmission of major parasitic zoonoses and promote bridging infections with domestic animals. Against this background, this article reports and discusses some of the most important endoparasites of wild mesocarnivores living in Europe, on the basis of their actual role as reservoirs, spreaders, or sentinels. The data derived from epizootiological studies in different European countries, and the proven or speculated implications of the detected endoparasites in human and domestic animals’ health, are discussed. Through older and recent literature review, the state-of-the-art knowledge on the occurrence and prevalence of the parasites under consideration is presented, showing further, warranted investigations and the need for surveillance and vigilance.

## 1. Introduction

The role of wild animals in the emergence and re-emergence of diseases, some of zoonotic concern, has been appreciated relatively recently [1]. It is typical that of all wildlife-derived zoonoses, those caused by viruses and bacteria gain most of the attention, probably because they may lead to dramatic epidemiological situations, epidemics, and pandemics. On the other hand, wildlife-derived zoonotic parasitoses usually cause less spectacular dispersion among populations, often remain asymptomatic, and due to their ways of transmission and complex life cycles, do not cause epidemics or pandemics. This may be why wildlife-associated zoonotic parasites receive less scientific and administrative attention and effort, in the frame of One Health alerts, approaches, and strategies [1]. However, human infections with parasites originating from wildlife have been identified and can be characterised as food-borne, vector-borne, and environment-borne.

Similarly, bridging infections between wild and domestic animals is a recognised phenomenon that seems to be occurring more often in recent years [2,3]. Such infections are influenced by climate change, land-use change, and novel human activities. Human exploitation of the environment led to a dissolution of natural barriers that used to function as disease control. As a result, wildlife, domestic animals, and humans share zones of sympatric habitats, where bridging infections may occur [4].

In Europe, such sympatric interactions often involve small- and mid-sized (<15 kg body weight) carnivore species, referred to by the term “mesocarnivores”. Mesocarnivores are represented by a much higher number of different species compared with large carnivores and display a variety of ecologies and behaviours. For example, these species can be solitary to highly social, may have a strictly carnivorous or an omnivorous diet and many of them are generalists regarding their habitat selection, thus they often live in close proximity to humans, occupying the top of the food chain, with no other animal species acting as a competitor. Their relatively small size and flexibility in thriving in various habitats result in the abundance of mesocarnivores in practically all kinds of environments [5]. Accordingly, wild mesocarnivores often develop their activity in proximity to humanised environments, and this has become more common in recent years, clearly because of the land-use changes, and the overall destruction and fragmentation of wild habitats and ecosystems, forcing wild animals closer to suburban and urban areas and, practically, to a territorial expansion or adaptation. This phenomenon is further enhanced by the introduction and proliferation of allochthonous species, such as the raccoon, the common racoon dog, and the American mink, which are among the most widespread invasive carnivores in Europe [6].

**Figure 1:**
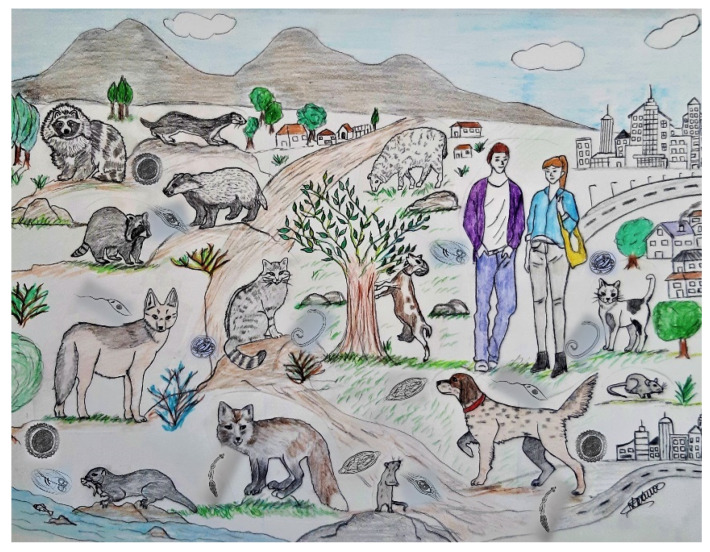
Wild mesocarnivores, domestic animals, and humans in sympatric settings can lead to the transmission of important zoonotic parasites and to bridging infections between wild and domestic animals.

The expansion and proliferation of mesocarnivores results, among other things, in bridging infections and transmission of parasites of wildlife origin to humans and domestic animals [1]. For example, *Baylisascaris procyonis*, a parasite of raccoons, is a recognised zoonotic nematode of emerging medical importance, and *Echinococcus multilocularis*, a taeniid of foxes, has infected humans with an increasing trend in Europe in recent years [1,7]. Similarly, these interactions lead to spillover parasitoses between related animal species, as evidenced in the case of *Angiostrongylus vasorum*, passing from its main host, the foxes, to sympatric dogs, and *Troglostrongylus brevior* which is common in domestic cats living in sympatry with wildcats, i.e., the natural definitive host of the parasite (Figure 1) [2,3].

This review aims to explore the role of wild mesocarnivores as reservoirs and bridge hosts of important emerging parasites in Europe, taking into consideration the distribution, biology, and behaviour of these animals, and providing some basic information about the impact of the parasites on human and domestic animals’ health.

The topic is approached on an animal-group or animal-species basis. Starting with the family of Canidae, arguably the most abundant species, the red fox (*Vulpes vulpes*), the golden jackal (*Canis aureus*), and the invasive common raccoon dog (*Nyctereutes procyonoides*) are addressed. The family Procionidae is represented by the invasive raccoon (*Procyon lotor*) and Felidae by the widespread European wildcat (*Felis silvestris*). Finally, from the family Mustelidae, the prolific genera *Meles*, *Martes*, *Mustela*, *Lutra*, and the invasive American mink (*Neogale vison*), are approached under the scope of the present article. A list of the parasites discussed and mentioned in each animal species in this article is presented in Table 1.

Some ideas on future strategies, based on surveillance and monitoring, regarding the prevention of further emergence of wildlife-derived parasitic zoonoses, are discussed.

## 2. Canidae

In Europe, the wild mesocarnivore members of the family Canidae consist of the red fox (*Vulpes vulpes*), the golden jackal (*Canis aureus*), and the raccoon dog (*Nyctereutes procyonoides*) [8]. The red fox and Golden jackal are indigenous species in Europe, with differences in the areas of origin and distribution. On the other hand, the raccoon dog is an alien species brought from Russia during the first half of the 20th century which then spread rapidly into many European countries (see File S1) [9,10].

### 2.1. Red Fox (Vulpes vulpes)

The red fox (from now on “fox”) is a very adaptable species with a generalist diet, high reproductive potential, and social flexibility [11]. These characteristics, in combination with the success of rabies control [12], allowed an increase in the fox population from the 1990s and a geographical expansion from traditionally endemic areas of central Europe to the east (i.e., western Russia and the Baltic states) and other European regions (see File S1) [13,14]. Alongside this, over time, there has been a progressive urbanisation of fox populations due to the fragmentation of their habitats, followed by easy adaptation to suburban and urban environments [15]. Typically, synurbic foxes reach higher population densities when compared with animals in wild habitats, favoured by sufficient and seasonally stable food of anthropogenic origin (e.g., waste or pet food) [16]. In urban and peri-urban areas, the frequency of contact between foxes, humans, and pets has changed from sporadic to constant, significantly increasing the chances of parasite transmission [17,18]. Parasites shared by foxes, pets, and humans in Europe have been previously reviewed [19,20]. Foxes represent the natural reservoir for various endoparasites traditionally showing a wildlife cycle, transmitted by the faecal–oral route or via intermediate hosts [20,21]. On the other hand, they act as dead-end hosts for some foodborne human infections, as in Europe fox meat is generally not consumed. However, they may represent an excellent indicator for the circulation of food-borne parasites, being nonspecific predators/scavengers, and widespread, contributing to the maintenance of parasites’ life cycle in wild settings [22,23].

In addition, they may be excellent sentinels of vector-borne diseases (VBDs), mainly because of their proximity to urban or agricultural settings and frequent exposure to different arthropod vectors (e.g., ticks, mosquitos, sand flies) for which they represent a good blood meal source [19,24,25].

#### 2.1.1. *Leishmania infantum*: The Role of the Red Fox Is Still Unclear

Leishmaniosis is a severe parasitic disease caused in Europe by the kinetoplastid protozoon *L. infantum*, transmitted by sand flies of the genus *Phlebotomus* [26]. The role of several wild canid species as potential primary or secondary reservoirs has been investigated due to their close phylogenetic relationship with the main reservoir, the dog.

A consistent exposition to *Leishmania* infection was demonstrated in fox populations in Europe, by serology and molecular surveys, with prevalence ranging widely [27]. The lowest prevalence values were detected in France (9–15%) [28,29] and Georgia (2.6%) [30] and the highest in the highly endemic regions of the Mediterranean Basin, i.e., Italy (6–50%) [31,32,33,34], Greece (59.5%) [35], and Spain (12–74%) [36], usually in line with those recorded in dogs in the same areas. However, the rates of parasite detection in the skin of foxes vary greatly, from 0% to 34% in highly endemic areas [32,33] and are much lower if compared to deep organs (i.e., spleen, bone marrow, or lymph nodes). These findings lead to the speculation that foxes might be less capable of infecting phlebotomine vectors than dogs, as dogs’ skin shows positive rates similar to or even higher than deep organs [37]. However, further studies are needed to elucidate the role of foxes in the epidemiology of *L. infantum*.

Entomological surveys conducted in Europe showed that foxes act as a good blood source for *Phlebotomus pernicious*, and frequently share an ecosystem with rabbits and hares that are proven amplifier hosts of the parasite [38,39]. However, to date, there have been no investigations regarding the infectiousness of foxes to sandflies (xenodiagnosis). In fact, studies carried out in Brazil showed that *Lutzomia longipalpis* (the main vector of *Leishmania chagasi* in the New World) did not become infected after blood meals on infected crab-eating fox (*Cerdocyon thous*) [40].

Most studies on foxes indicate that infected animals do not present any clinical signs. However, in Greece, the majority of the animals examined (63.8%) showed at least two to three clinical signs compatible with canine leishmaniosis (CanL), including low weight, dermatitis, skin lesions, alopecia, splenomegaly, enlargement of lymph nodes, and onychogryphosis [35].

#### 2.1.2. *Babesia* spp. and *Hepatozoon* spp.: The Red Fox as a Competent Reservoir

Red foxes have been identified as reservoirs for tick-borne pathogens (TBPs), including hemoprotozoan organisms of the genera *Babesia* and *Hepatozoon* [41].

In Europe, *Babesia canis* is the main species infecting dogs, and although it has also been reported in foxes, these animals do not seem to act as a reservoir of the parasite [42]. A second species causing babesiosis in dogs, i.e., *Babesia vogeli*, was also reported in red foxes in France [43].

However, red foxes have been proposed as the natural host of *Babesia vulpes* (syn. *B*. *microti*-like piroplasm, *Babesia* sp. “Spanish dog”, *Theileria annae*) [44,45], due to their high infection rates and the absence of clinical signs in most of the cases. The effective tick vector of *B. vulpes* is still unknown [46], but *Ixodes hexagonus*, a triphasic hard tick with esophilic behaviour, has been speculated as a potential vector [47].

The highest prevalences of fox infection with *B. vulpes* in Europe are recorded in Portugal and Spain, where 68% to 100% of animals are infected, depending on the sample nature (blood or bone marrow) and the analysed geographic area [48,49]. The presence of *B. vulpes* at high prevalence was also described in Germany (46.5%) [50], Austria (50.7%) [51], and Italy (5–69.2%) [52,53], while in other countries, the prevalence is lower, e.g., 20.7% in Romania [54], 20% in Hungary [55], 32% in Bosnia and Herzegovina, [51], 30.7% in Poland [46], and 5% in Croatia [56]. The pathogenic consequences of *B. vulpes* in red foxes have not been thoroughly studied. However, enlargement of both kidneys and the spleen has been observed in necropsied infected foxes [46].

*Hepatozoon canis* is commonly detected in European fox populations. *Hepatozoon* infection is acquired through the ingestion of an infected ixodid tick. In southern Europe, *Rhipicephalus sanguineus* sensu lato (s.l.), widely distributed and strongly associated with dog presence, is considered the main vector of the parasite [57]. However, in areas where *R. sanguineus* s.l. is not endemic (e.g., Austria, Slovakia, Germany, Poland), the role of alternative species, e.g., *Ixodes ricinus* and *Dermacentor reticulatus* as competent vectors has been speculated [42]. Alternative routes of *H. canis* transmission are transplacental, proven in both foxes and dogs [51], and the consumption of infected prey carrying *H. canis* tissue cysts [58].

A high prevalence of *H. canis* has been detected in fox populations from central and southern Italy (50%) [34,52], Germany (45.2%) [50], and Poland (44.7%) [46], with higher values in Portugal (75.6%) [59], Serbia (61.2%) [60], and the Czech Republic (95%) [61], while lower rates were recorded in Bosnia and Herzegovina (38.6%) [51], Croatia (23%) [56], Austria (18.5%) [62], Slovakia (17.1%) [63], and Hungary (8%) [64]. Hepatozoonosis in foxes, similar to dogs, generally develops as a subclinical infection; however, a slightly enlarged spleen and liver may be observed [46].

#### 2.1.3. *Dirofilaria immitis* and *Dirofilaria repens*: Red Foxes Are Infected but Not Quite Infectious

The fox has been proven able to act as a definitive host for both *Dirofilaria immitis* and *Dirofilaria repens* (the causative agents of canine cardiopulmonary and subcutaneous dirofilariosis, respectively) but infections in foxes are generally regarded as an epiphenomenon of the dog infection in overlapping habitats [19]. Since foxes tend to show lower levels of heartworm infections (the burden of *D. repens* infection is more difficult to be estimated) and lower microfilaremia compared to dogs [65], it has been speculated that they are not an epidemiologically important host for competent vectors and thus they are of minor significance from an epidemiological and a public health perspective [66].

In the traditional endemic areas of southern Europe, infection in foxes is reported with prevalences between 1.5% and 30% for *D. immitis* and from 1.2% to 22% for *D. repens* [43]. However, the presence of filarial infections in low endemic areas, i.e., Hungary [67], Romania [68], Russia [69], and Serbia was also reported with prevalences between 0.3–8.5% and 0.7–6.4% for *D. immitis* and *D. repens*, respectively [70].

To date, the clinical impact of these two major filarids in foxes is unknown and deserves future investigation.

#### 2.1.4. *Thelazia callipaeda*: Red Foxes as an Important Source of Infection

*Thelazia callipaeda* is a vector-borne zoonotic nematode with the zoophilic fruitfly *Phortica variegata* as the only confirmed vector in Europe [71]. *Thelazia callipaeda* lives in the conjunctival sac and associated tissues of a relatively large spectrum of hosts, including carnivores, lagomorphs, and humans [72].

The importance of foxes in the epidemiology of *T. callipaeda* in different habitat types across Europe has been documented by the high prevalence of infection and the speculated frequent contact of foxes with the vector, favoured by the co-occurrence of the seasonality and crepuscular activity of *P. variegata* and fox activity patterns [51,73,74].

*Thelazia callipaeda* is endemic in fox populations of Italy, France, and Switzerland, but has also been expanding to various hosts in regions of Portugal, Greece, and Balkan areas [51,75,76]. The prevalence rates vary within large limits, from low, i.e., 5.1% and 5.7% in northern Italy and Switzerland, respectively, [77,78] to medium, i.e., 27.7% and 29.4% in Bosnia and Herzegovina, and Romania, respectively, [51,74] and high, i.e., 49.3% in southern Italy [79]. The occurrence of a unique genetic haplotype (h-1) of *T. callipaeda* in all specimens examined from foxes and pets suggests that this parasite circulates among domestic and wild carnivores in all areas where it has been reported [72].

The zoonotic potential of *T. callipaeda* is of concern mainly in rural areas of southeast Asia [80,81], however, ophthalmologists and general physicians should be particularly alert to possible human cases in all areas where the infection is known in foxes and other hosts [82]. In fact, sporadic human cases in Europe have been diagnosed in endemic countries, i.e, Italy, France [83], Spain [84], Serbia [85], Germany [86], Croatia [87], Portugal [88], and Hungary [89].

#### 2.1.5. *Toxoplasma gondii* and *Trichinella* spp.: The Red Fox as a Sentinel of Occurrence

*Toxoplasma gondii* and *Trichinella* spp. are food-borne pathogens of worldwide importance in human and veterinary medicine. Foxes do not serve as a direct source of human infection in European countries; however, they represent an excellent sentinel for the circulation of these parasites, which are typically present in predatory and scavenger wild carnivores [90]. Furthermore, foxes may have an active role in the maintenance and propagation of these parasites in the sylvatic cycle.

*Toxoplasma gondii* (see Section 4.1.1) can infect foxes through ingestion of sporulated oocysts (e.g., via contamination of food, water, soil), but more frequently, via predation of infected prey harbouring tissue cysts. Vertical transmission has also been proposed, as in other hosts, but there is no clear evidence of this [91].

A high level of fox populations exposition to the parasite is documented as a result of the cumulative effect of consuming infected prey. The highest *Toxoplasma* seroprevalence in foxes is detected in central eastern countries reaching up to 35% in Austria [92], 75–85% in Germany [93], and 100% in the Czech Republic and Belgium [94,95].

Genotyping of *T. gondii* isolates in red foxes has been performed only in a few studies, showing that most of the isolates belong to the clonal type II or mixed clonal type II and III [93,96] that represent typical archetypal lineages of the anthropogenic food sources [97].

Only scarce information is available regarding the clinical impact of *T. gondii* in foxes; however, apparently red foxes tolerate *T. gondii* infection very well in the absence of immunocompromising factors (i.e., canine distemper virus (CDV), vulpine circovirus (FoxCV)), compared with other fox species, i.e., blue foxes (*Vulpes lagopus*). In fact, experimental studies demonstrated that when oocysts or tissue cysts of the highly pathogenic strains GT-1 or TC-1 are fed to red fox puppies, the animals do not develop any clinical signs [98], contrary to experimentally infected blue foxes (*Vulpes lagopus*), which develop severe clinical toxoplasmosis after parenteral infection with tachyzoites of the highly virulent RH strain [99].

Interestingly, recent studies suggest that *T. gondii*, due to its neurotropism and the predilection for specific sites, is able to manipulate intermediate host behaviour in order to enhance transmission (i.e., limbic system) [100] and to trigger aberrant behaviour in foxes. An association between toxoplasmosis and a range of aberrant behavioural traits known as “dopey fox syndrome” (DFS), was speculated [101].

Of the 12 nematode species that comprise the genus *Trichinella*, 4 (*Trichinella spiralis*, *Trichinella britovi*, *Trichinella nativa*, and *Trichinella pseudospiralis*) circulate in Europe, showing different host ranges, geographical distributions, ecology, and histopathological features (i.e., encapsulated/not-encapsulated larvae) [102]. *Trichinella* spp. Has a peculiar monoxenic life cycle: carnivorous, omnivorous, and occasionally herbivorous animals are identified as hosts of the parasite, whose transmission occurs through the ingestion of infected meat containing the first larval stage [102]. Adult nematodes in the intestine produce larvae which migrate within the skeletal and cardiac muscles, causing clinical disease in susceptible hosts (e.g., humans).

A wide range of carnivores can harbour infective larvae of *Trichinella* spp., but foxes are considered the main reservoir of the sylvatic cycle and reflect the parasite’s presence in the food web since they are cumulatively exposed to infection due to their food habits [103]. There is contradictory information in scientific papers regarding the distribution of *Trichinella* larvae in the muscles of naturally infected foxes; however, a number of studies showed that the highest infection intensity is usually recorded in muscles of the trunk and head (i.e., masticatory muscle, diaphragm, intercostal muscles, and tongue root muscles) [104].

The prevalence of *Trichinella* spp. in fox populations varies significantly in European countries, with the lowest values (0.9–5%) observed in southern and western Europe and the highest (up to 40%) in the endemic northeastern regions (i.e., Finland, Serbia, Romania, Bulgaria, and Russia) [105,106]. In most cases, *T. britovi* has been identified in foxes, and only sporadically *T. spiralis*, suggesting a major role of the fox as a reservoir of *T. britovi* (sylvatic cycle), and a lower importance in the epidemiology of *T. spiralis* in the domestic cycle [107].

#### 2.1.6. *Echinococcus multilocularis*: The Red Fox Is the “Usual Suspect”

*Echinococcus multilocularis* is a small tapeworm of the Taeniidae family with a typical prey–predator life cycle [108] that develops predominantly in sylvatic environments and rural settlements. The adult parasite lives in the small intestine of wild carnivores (definitive hosts), producing mature proglottids with eggs that are released into the environment via the faeces. Small rodents of the family Arvicolidae and Muridae (e.g., *Microtus arvalis*; *Apodemus* spp.) act as intermediate hosts and become infected by ingesting (e.g., via food or water) the eggs of the parasite. The larval (metacestode) stage of the parasite, the alveolar cyst, develops in the liver and other internal organs of the intermediate host [108,109]. In Europe, the fox is considered the main definitive host of *E. multilocularis*, and together with raccoon dogs, is used as sentinel by the monitoring system of the EFSA Council [110].

Historically, endemic regions for *E. multilocularis* in Europe consisted of a ‘core’ area including southern Germany, eastern France, north-central Switzerland, eastern Italy, and western Austria, where prevalences in foxes ranges from 15–65% [7,111,112]. However, broad epidemiological studies reported a significant expansion of the parasite to northern and eastern countries, i.e., Denmark, Sweden, Lithuania, Estonia, and Poland [7,113], with peaks observed in Estonia (29.4%) [114], Latvia (35.6%) [115], and Lithuania (58%) [109].

*Echinococcus multilocularis* is the causative agent of alveolar echinococcosis (AE) in humans, a serious disease that can be fatal if left untreated [109]. The prevalence of human AE cases in Europe is approximately proportional to the prevalence of infection in the definitive hosts (mainly foxes) and displays a significantly increasing trend since 2010 [116]. Moreover, human cases of AE are being reported more frequently in urban areas, as a consequence of the urbanization of the *E. multilocularis* life cycle, due to the approach of foxes to the peri-urban/urban environment [19]. Although *E. multilocularis* prevalence in foxes in such environments is usually lower than in rural and wild settings, because foxes consume a lower proportion of intermediate hosts, the enlarging fox densities in urban settings may enhance the risk of bridging and zoonotic infections in these particular areas [117].

In Europe, surveys revealed that adult foxes, due to partial immunity acquired after exposure, display a lower prevalence of infection and lower worm burdens than foxes under one year of age, rendering young foxes the main drivers of infection spread [118]. This finding is different for *T. gondii* and *Trichinella* spp. for which adult animals show a higher prevalence of infection due to the long life of these parasites in host tissues that remain viable for many years (*Trichinella* spp.) or even lifelong (*T. gondii*) after the first infection of the host [119].

#### 2.1.7. *Angiostrongylus vasorum* and Airway Capillarioses: Red Foxes Contribute to Their Expansion

The fox is considered the major reservoir host and spreader of *Capillaria aerophila* (syn. *Eucoleus aerophilus*) and *Capillaria boehmi* (syn. *Eucoleus boehmi*), the agents of the lung and nasal capillariosis, respectively, and Angiostrongylus vasorum, also known as French heartworm [19,21,120,121,122]. In the last two decades, these parasites have been recognised as emerging agents of disease in dogs in several European countries [122].

The capillarids of the airway are typical soil-transmitted nematodes, with a direct cycle: immature eggs are excreted with the faeces of the definitive hosts, and the infective L1 develops inside within 15–40 days for *C. boehmi* and 35–60 days for *C. aerophila* [123]. Infection is acquired through the ingestion of embryonated eggs, while earthworms are suggested to act as facultative intermediates or transport hosts [123].

*Capillaria aerophila* and *C. boehmi* share common patterns of transmission and thus are frequently detected in co-infections. Foxes show rates of positivity that vary a lot (from 4% to 100%) among different geographical European countries. More precisely, *C. aerophila* infection in foxes reaches 97% in Lithuania, 88.8% in Norway, 84% in Serbia, 76.2% in Poland, 75% in Germany, 66% in Hungary, and 65% in the Netherlands [124,125,126,127,128,129,130,131]. The *Capillaria boehmi* infection rate in foxes ranged from 8% in Hungary [130] to 30.7–51% in Italy and Norway [21,126,132], whereas the highest prevalence (90%) has been recorded in Serbia [127].

Few cases of human infection by *C. aerophila* mimicking the clinical and radiographic findings of pulmonary bronchial carcinoma have been described in Europe, i.e., Russia, Ukraine, France, and Serbia [127,133,134], where the prevalence of infection in foxes is high (up to 84%).

*Angiostrongylus vasorum* is a metastrongyloid nematode characterised by an indirect life cycle: definitive hosts (domestic dogs and wild canids) acquire the infection by the ingestion of infected gastropods harbouring the infective third-larval stage (L3) or of paratenic hosts, such as frogs, birds (e.g., *Rana temporaria*, *Gallus gallus* domesticus), and other small vertebrates [135,136,137].

*Angiostrongylus vasorum* was traditionally endemic in southwestern France, the UK, and Denmark [138], where infection in dogs was documented in isolated foci with only sporadic occurrence outside those areas. However, over the last 20 years, an expansion of the parasite’s geographical range and spreading to new geographical areas (e.g., Italy, Bulgaria, Slovakia, Poland, Serbia, Belgium, Romania) has been documented [122,139,140]. The decisive factors of this expansion include growing fox populations, the movement of dogs from endemic to non-endemic areas, and climate changes impacting the intermediate hosts’ epidemiology, as observed in other snail-borne diseases [141,142]. After the first detection of *A. vasorum* in British foxes [143] a number of epidemiological surveys were conducted in these animals and variable rates were recorded with higher prevalence found in central Italy (75–78%) [132,144] and north-eastern Switzerland (81.8%) [145], followed by Great Britain, Hungary, Norway, Poland, Republic of Ireland, Romania, Serbia, Slovakia, Spain, and the Netherlands, with rates up 43% [144].

Unlike dogs, in which *A. vasorum* infection may show a wide spectrum of clinical manifestations, ranging from no or minor clinical signs to life-threatening disease [146], foxes usually exhibit chronic infections with mild clinical signs that occur when concurrent infective diseases and high worm burdens are present [141,147]. However, fatal infections in foxes have been recently described [148].

#### 2.1.8. Other Parasites

Foxes may harbour various parasites in addition to those mentioned above, which may be shared with pets and/or have zoonotic implications.

Foxes may get infected with cyst-forming parasites further than *T. gondii* by prey or carrion consumption, especially mice and deer; in fact, foxes may act both as intermediate and definitive host for *Neospora caninum*, despite the fact that to date, no active oocyst shedding has been described in foxes in Europe. Biomolecular and serological surveys showed low to moderate prevalences of *N. caninum* infection in foxes in Europe, lower than those recorded for *T. gondii* [95].

Low to medium rates of positivity for *Cryptosporidium parvum* and *Giardia duodenalis* have also been detected [149,150]. Foxes may also show patent infections through a wide range of tapeworms of the genus *Taenia*, especially those transmitted through predation of rodents or rabbits/hares, i.e., *Taenia crassiceps*, *Taenia pisiformis* [151,152], and *Mesocestoides* spp. [153]. On the other hand, *Echinococcus granulosus* s.l. is very rarely detected in foxes and usually with low burdens [19].

Foxes are also suitable definitive hosts for various species of trematodes. Among those, the most important in terms of frequency and public health implications are the meat-borne intestinal fluke *Alaria alata* [154] and the fish-borne liver fluke *Opisthorchis felineus* [90].

Typical soil-transmitted nematodes in dogs, e.g., *Trichuris vulpis*, *Toxocara canis*, and *Uncinaria stenocephala* may equally have foxes as natural hosts [20,155,156]. In contrast to the high prevalence observed for *U*. *stenocephala*, a scant detection of the other ancylostomatid, *Ancylostoma caninum*, is recorded [20].

Among the extra-intestinal parasites, *Crenosoma vulpis* and *Capillaria plica* are also being recorded at high prevalences in foxes [157] and a new species of the genus *Spirocerca*, i.e., *Spirocerca vulpis*, is emerging in foxes from Bosnia, Herzegovina, Italy, and Spain [158]. Interestingly, *S*. *vulpis* exhibits a different nodule location, focused on the stomach wall compared to the most known *Spirocerca lupi*, which mainly develops in the oesophagus and aorta of dogs and wolves [159].

### 2.2. Golden Jackal (Canis aureus)

At the beginning of the century, the population of the Golden jackal (from now on “jackal”), also known as the common or Asiatic jackal, Eurasian Golden jackal, or reed wolf [160,161], in Europe declined dramatically; however, a notable expansion started in the 1950s, allowing a progressive recolonisation in Europe [162,163]. Currently, stable, reproducing populations with high densities are being recorded in approximately 20 countries in central and eastern Europe, while in others, sporadic vagrant animals are observed (see File S1) [164]. The factors favouring the territorial expansion of jackals are unclear, but land use, climate change, and the lack of natural predators have been suggested [165].

The jackal has an opportunistic nutritional behaviour with an extremely varied diet consisting mostly of small mammals, birds, and their eggs, but is also a scavenger and is capable of using anthropogenic food sources [162]. This wide food spectrum, their relatively broad territorial mobility (from 1 to 20 km^2^), and their ability to adapt to novel habitats both in suburban and urban areas allow jackals to come into contact with animals that live in close proximity to humans, such as dogs and foxes [162]. With this in mind, taking into account that jackals act as hosts to a wide variety of canine and zoonotic parasites, it should be considered that they may play an important role in bridging infections to domestic animals and humans [162,166].

#### 2.2.1. *Dirofilaria immitis* and *Dirofilaria repens*: Golden jackals Are Suitable Spreaders

Jackals are considered a potential wild reservoir of filaroids in endemic areas of Europe, particularly for *D*. *immitis*, as they are frequently found infected both in necropsies (adult worms) and in blood examinations (microfilariae), and excessive microfilaremia are recorded in up to 50% of infected animals [19,68,167]. On the contrary, *D. repens* has been found in the blood of jackals by molecular methods at a low prevalence (3%) [68].

In endemic areas, the spatial distribution and prevalence of *D. immitis* infection are similar to those recorded in dogs, suggesting that jackals might play a role in the transmission and the maintenance of the parasite comparable to dogs, i.e, the primary definitive host [68]. In fact, unlike dogs, jackals are out of any preventative control and represent a good blood source for *Aedes albopictus* and *Culex pipiens* complex mosquitoes, which are the most efficient vectors of *D. immitis* in Europe [68]. The highest prevalence of *D. immitis* infection in jackals is recorded in the Balkans and central European countries, e.g., Bulgaria, Serbia [168], Hungary [67], and Romania [68], where it reaches up to 35% of animals.

#### 2.2.2. Other Parasites

Jackals can be exposed to several vector-borne pathogens that primarily affect dogs, suggesting the hypothesis of bridging infections. For instance, *L*. *infantum*, *B*. *canis*, and *H. canis* have been found at the margin of the endemic areas of CanL and canine babesiosis [162] and to date they are the only species isolates in jackals in Europe.

From the public health impact point of view, jackals should be considered a natural sylvatic reservoir of *Trichinella* spp. The most common species isolated in this animal species is *T*. *britovi*; however, in the Balkans *T. spiralis* has also been identified [169]. Since vagrant individuals may migrate for long distances, they may contribute to the introduction and spread of this parasite in neighbouring countries where *Trichinella* spp. is less prevalent (i.e., Greece, Italy, and northern Macedonia).

Jackals may also serve as the definitive host for both *E. granulosus* and *E. multilocularis* [109]. In fact, this wild canid frequently preys on rodents, including arvicolins and other easily available food sources, such as viscera and carrions that may promote infection with *Echinococcus* spp. [170]. In the European continent, the fox and the domestic dog remain the major definitive hosts of *E. multilocularis* and *E. granulosus*, respectively, (see Section 2.1.5) and jackals and other wild canids, i.e., the raccoon dog, have an inferior importance in the sylvatic and domestic life cycles of the parasites, in particular of *E*. *granulosus* [171]. However, they have been identified as definitive hosts for *E. granulosus* in Hungary, Bulgaria, and Italy [166]. There is a general agreement, that in central and eastern European regions where *E. multilocularis* prevalence is high in foxes, a correlated low to moderate infection prevalence (up to 5%) can be also observed in secondary definitive hosts such as jackals, raising a question on the role of this wild canid as an additional, albeit minor, reservoir and infection source for humans [109,172,173]. In fact, *E. multilocularis* was detected in jackals from Hungary, Serbia, Croatia, Austria, and Switzerland [166].

The presence of traditional soil-transmitted nematodes is described in jackal populations in Europe but with low to moderate prevalences compared to other wild canids (i.e., wolf, foxes, raccoon dogs) [162]. However, a relatively prevalent presence of tapeworms, i.e., *Dipylidium caninum*, *Multiceps multiceps*, and *Mesocestoides* spp. is documented [162]. A widely distributed trematode in jackals is *A*. *alata*, found in Russia and in the Balkans [174].

Finally, jackals have also been found to be infected with a wide range of extra-intestinal nematodes, i.e., *A*. *vasorum*, *S*. *lupi*, *C*. *plica*, *C*. *aerophila*, and *T*. *callipaeda*, but the reports are scant [162,175]. In Europe, *T. callipaeda* was found only in one jackal in Romania, with high intensity of infection (70 nematodes) [175]. The absence of similar findings in other areas is probably due to a lack of full post-mortem examination (e.g., when the eyes are damaged) and thus it cannot be excluded that jackals may act as a potential reservoir host of this parasite.

### 2.3. Raccoon Dog (Nyctereutes procyonoides) 

The raccoon dog originates from east Asia and is one of the most successful invasive carnivores in Europe. It was introduced in the western Soviet Union for its fur in the middle of the 20th century [176,177]. The successful control of rabies from the 1990s, together with the high reproductive capacity and behavioural adaptation of this species, led to its successful establishment and expansion all over Europe (see File S1) [13,178]. One of the major threats arising from the growing and expanding population of raccoon dogs is linked to their role in maintaining and transmitting various parasites, some of high zoonotic concern, for which raccoon dogs may act as important definitive hosts in wild environments [13,179,180].

#### *Trichinella* spp., *Echinococcus multilocularis* and Other Parasites

The raccoon dog is a suitable indicator and a well-adapted host for all four species of the genus *Trichinella* circulating in Europe (see Section 2.1.5) [181], which can be detected in single or mixed infections. This wild canid plays an important role, especially in spreading *T. spiralis* and *T. britovi* through the sylvatic cycle in north-eastern European countries [182,183]. The prevalence of *Trichinella* infection in raccoon dogs varies widely between different European regions, from 0% in Denmark and Austria, to 1.9–4% in Germany, 33.2% in Finland, 35.5% in Lithuania, 32.5% in Latvia, and 57.5% in Estonia [176,184,185,186,187].

Although little information is available on the predilection of raccoon dog muscles to *Trichinella* infection, an experimental infection with *T. spiralis* and *T*. *nativa* showed that the highest larval loads were detected in the foreleg, eye, and tongue muscles of the animals [188].

The raccoon dog has been documented as an additional definitive host for *E. multilocularis* in Europe, as it is highly susceptible to this parasite [109,189,190]. With the exception of Slovakia, where *E. multilocularis* prevalence in raccoon dogs is ~27% [191], i.e., similar to that recorded in foxes [105], in countries of high endemicity (Germany, Poland, Czech Republic, Slovakia, Switzerland, Austria, and Lithuania), these animals are less frequently infected than foxes, with prevalences ranging from 1.6% to 8.2% [180,192]. The different feeding preferences of the raccoon dog (amphibians) compared to fox (rodents) could be the reason for the prevalence difference of such taenidae [178]. Actually, although the raccoon dog may act as a definitive host for *E*. *multilocularis*, there is no evidence that it could maintain the life cycle of the parasite in the absence of the main reservoir, i.e., the fox [189].

A parasite detected in raccoon dogs with an increasing frequency in some European countries is *A*. *alata*, with a prevalence from 30% in Austria [186] to 96.5% in Lithuania [151,178]. The adult stage of this trematode has been found in the intestine of raccoon dogs with little pathogenic relevance; however, the larval stage (mesocercariae) found in paratenic hosts, e.g., wild boars (*Sus scrofa*), is pathogenic and represents a problem of public health concern [193].

## 3. Procionidae

The mesocarnivores of the family Procionidae include several mammals native to North and South America [194]. The raccoon (*Procyon lotor*), represents the only procionid established as an alien species in several European countries and areas of the Caucasus [195].

### 3.1. Raccoon (Procyon lotor)

The raccoon was introduced into Europe and Asia in the early 20th century, where it was released for hunting purposes or escaped or was set free from captivity (i.e., fur farms, houses) [196]. Despite the fact that most of the raccoon populations in Europe do not reach high densities, over 100 animals per km^2^ have been detected in some peri-urban areas of Germany, i.e., the country hosting the largest population outside America [183,197]. Stable populations are currently developing in Spain and France and some newly established populations are present in many central, northern, and eastern European countries (see File S1) [198,199]. Te raccoon’s success as an introduced species is attributed to its adaptability to different environments, omnivorous feeding habits, high reproductive potential, and the lack of natural predators [199,200].

One of the major threats arising from the growing and expanding population of raccoons in Europe is related to pathogens that are transmittable to humans and domestic animals [200]. Raccoons in Europe seem to have an impoverished parasitic fauna compared with those of the North American populations, both in terms of species diversity and abundance of parasites, although they have acquired a few parasite taxa endemic in Europe [201]. In any case, raccoons represent a potential source of exotic parasite spillover to other wild animals, dogs, and humans.

#### *Baylisascaris procyonis*, *Strongyloides procyonis* and Other Parasites

*Baylisascaris procyonis* is an ascarid nematode, characterised by its particular zoonotic importance and severe implications for human health [202]. Raccoons and related procionids are the main hosts of this parasite, which can also develop to the adult stage in dogs [203]. It is a typical geohelminth, acquired through oral–faecal transmission from the environment. The infected host sheds millions of eggs daily that become infective (containing the third-stage larva, L3) in approximately 10–12 days and remain infective for months or years [204]. In Europe, *B. procyonis* infection in raccoons has been documented in over 70% of raccoons in central Germany [196,205] and in 33.3% in Italy at necropsy [206]. Raccoons infected with *B*. *procyonis* usually do not exhibit clinical signs [203]. However, heavy burdens have been associated with intestinal obstruction [207,208].

A wide range of mammals, including humans, may accidentally ingest infected eggs from the environment with subsequent health implications. Raccoons, adapted to peri-urban and urban areas, defecate at latrines close to their resting places in peri-domestic sites, e.g., barns, lofts, attics, chimneys, or garages [196]. This typical behaviour increases the risk of human and dog exposure to *B*. *procyonis* infection [209].

When humans accidentally ingest the infected eggs, L3 hatch and may start their plurivisceral migration, causing ocular and visceral larva migrans syndrome (OLM, VLM), which may become fatal when larvae invade the central nervous system (neural larva migrans syndrome, NLM), causing eosinophilic meningoencephalitis with progressive neurological disorders [210]. Human baylisacariosis is an emerging zoonosis, usually described in patients (often children) who had close contact with these animals [202,211]. In Europe, human baylisacariosis has been documented only once, in Germany, in a patient who had purchased a raccoon from a local zoo and presented with unilateral neuroretinitis syndrome [212].

Another parasite strongly associated with raccoons is *Strongyloides procyonis*, a threadworm infecting mainly the small intestine. The biology of *S. procyonis* is poorly known, and presumably similar to that of *Strongyloides stercoralis*, in which infective filariform larvae from the environment enter the host through transdermal penetration and follow the tracheal migration until they reach the small intestine where they mature into female adults that produce eggs through parthenogenesis. Larvae hatch from eggs directly in the intestinal mucosa, initiating autoinfective cycles with a remarkable persistence within the host [213].

The first descriptions of *S. procyonis* in raccoons are from North America [214], while to date, in Europe the parasite was found in raccoons in Poland [215] and recently in central Italy, where a parasitological investigation showed a prevalence of 26.9% [201]. Susceptibility of dogs to *S. procyonis* has been experimentally demonstrated [216], as well as that of humans, through a healthy volunteer who developed a short-lived intestinal infection [217]. However, *S. procyonis* in humans and dogs may be confused with the morphologically similar, most common species *S. stercoralis* [218], resulting in an overall underestimation of *S. procyonis* occurrence. Such an underestimation could also be favoured by the fact that the detection of parasitic females of *S. procyonis* is not easy, even after a thorough inspection of the mucosa scraped from the whole length of the intestine.

The emergence of this exotic parasite does not appear particularly alarming from a public health point of view, as it is known to cause at most mild dermatitis in humans [217]. However, the course of infection in immunocompromised individuals or animals remains unknown, as well as the impact on naïve native mesocarnivores (e.g., mustelids) that share habitats with raccoons. To properly assess the potential health threats to indigenous domestic and wild species, field surveys as well as laboratory characterisation of the sparsely known biology of *S. procyonis* are necessary.

Raccoons may also be rarely infected with other endoparasites shared with domestic and wild canids, i.e., *Leishmania* spp., *D*. *immitis*, *T. gondii*, and *Trichinella* spp., but their role in the transmission and epidemiology of these parasites is insignificant or still not well defined in Europe as in raccoons’ native countries.

## 4. Felidae

The wild members of the Felidae family in Europe are the Eurasian lynx (Lynx lynx), the Iberian lynx (*Lynx pardinus*), and the European wildcat (*Felis silvestris*) [219]. Both *Lynx* species are classified into the large carnivores of Europe [220], leaving the European wildcat the only representative of the family in the group of wild mesocarnivores [221].

### 4.1. European Wildcat (Felis silvestris)

The European wildcat (from now on “wildcat”) is widespread in Europe, with its largest population in the eastern-central, eastern, and south-eastern continent (see File S1) [222]. Although the species is assessed as least concern (LC) by the International Union for Conservation of Nature (IUCN), and despite the limited reliable data on the trends of its range and size, the populations are estimated to be declining in most areas, with an overall fragmented distribution [222]. The fragmentation and destruction of wildcat habitats are among the reasons for the observed declining population and results in forcing the animals closer to human settlements and domestic animals. This is an important drive for spreading wildcat parasites further from the sylvatic cycles to the margins of wild and humanised environments, rendering spillovers a realistic scenario [3]. Wildcats host a great number of different parasites [223,224,225,226], but in this article, some of the most important, in terms of bridging infections and public health, are presented.

#### 4.1.1. *Toxoplasma* gondii: The European Wildcat Is a “Master of the Game”

Felids have the “privilege” of maintaining the circulation of the protozoan parasite *T. gondii* in nature as the only definitive hosts, developing the enteroepithelial phase of the parasite’s life cycle and shedding oocysts to the environment via their faeces [226]. Wildcats were confirmed as definitive hosts of *T. gondii* approximately 30 years after the discovery of the parasite’s lifecycle [227,228]. A great range of vertebrates serve as intermediate hosts, including humans, and interestingly the infection has even been documented in species living in environments where the definitive hosts are not present, e.g., in polar bears (*Ursus maritimus*) in the Arctic [229]. In intermediate hosts, but in felids as well, the parasite develops tissue cysts. Both definitive and intermediate hosts may be infected by three routes of transmission: by ingesting sporulated oocysts from the environment (soil, water), by consuming raw meat with tissue cysts, and vertically, i.e., transplacentally or lactogenically [230]. *Toxoplasma* infection is usually asymptomatic in felids, but clinical disease may occur in other animals and humans. Accordingly, *T. gondii* is an important abortive agent in small ruminants and the cause of neurological disorders in many other species [231]. Infections with occasionally fatal outcomes have been reported in many wildlife species, e.g., red foxes, mustelids and other carnivores [230], red squirrels [232], and lagomorphs [233]. In humans, clinical toxoplasmosis usually occurs in immunocompromised patients, and it is also detrimental in congenital infections, causing encephalitis, retinochoroiditis, multiple organ damage, and death [231].

Infection in wildcats in Europe has been documented in a number of surveys. A total of 5 out of 6, 3 out of 6, and all 23 examined wildcats were found *T. gondii* seropositive in southern Portugal, Spain, and Great Britain, respectively [234,235,236], while in a wider serosurvey in France, 75 out of 112 wildcats (67.0%), were found seropositive [237]. Molecular detection of the parasite was successful in 4 out of 12 wildcats examined in Germany [238]. According to a recent systematic review and meta-analysis of the literature, the wildcat is ranked only second to the lion (*Panthera leo*) in terms of *T. gondii* seroprevalence [239].

Among the spreaders of *T. gondii* in Europe, i.e., the felids, wildcats hold an important position because of their wide geographical dispersion, their proliferation in various environments, and their high frequency of infection due to their predatory nature. Although it was evidenced that wildcats are infected equally prevalently as stray rural cats in France [237], other studies show an impressively high, and in some cases universal infection of the examined sample [234,236]. This is not surprising, as wildcats are exclusively fed by prey and have no access to, nor preference for, human-made food, a behaviour resulting in a higher prevalence of parasitic infections and polyparasitism compared to domestic cats, even stray ones [240]. For this reason, wildcats hold a key role in the circulation of the parasite in sylvatic and rural-suburban environments and subsequently in the infection of other wild and domestic animals, and humans. The risk of human infection at the conjunction of the sylvatic and domestic cycles is enhanced by the fact that the market and the trend for game meat consumption is currently under development, as consumers turn to more healthy and natural food options [154]. Tissue cysts of *T. gondii* in the meat of wild animals, e.g., hares, cervids, and wild boars, is a source of human infection when consumed raw or undercooked, while it has also been evidenced that people handling game meat (evisceration, skinning) are also at risk of infection when proper hygiene measures are overlooked [91].

#### 4.1.2. The Haemoparasites *Hepatozoon* spp., and *Cytauxzoon* spp.

The apicomplexan parasites *Hepatozoon* spp. and *Cytauxzoon* spp. are blood-associated TBPs that can infect wild and domestic felids. The feline hepatozoonosis and cytauxzoonosis vectors are unknown and various hard tick species may be involved [241]. Wildcats and stray domestic cats are more prevalently infected than domestic cats that live indoors, as they are more frequently exposed to tick vectors. In fact, wildcats are important for the maintenance of the sylvatic cycle of VBPs and likely play an important epizootiological role in spillover infections to domestic animals [241].

Feline hepatozoonosis is typically subclinical, but fatal myocarditis due to *H*. *silvestris* in a domestic cat has been recently reported [242]. Wildcats and domestic cats share two different *Hepatozoon* species (*Hepatozoon felis* and *Hepatozoon silvestris* [243,244,245]) while molecular and phylogenetic analysis support the hypothesis of a species-complex classification for *H*. *felis* [246,247]. *Hepatozoon* spp. is transmitted when the host ingests a tick containing the infective oocysts of the protozoan [248]. This particularity in transmission compared to the typical VBPs transmission, i.e., by a vector blood meal, is associated with the high prevalence of *Hepatozoon* infections in felids due to grooming behaviour, and also to infection through ingestion of parasite cystozoites in prey tissues [246,249]. Thus, because of their exclusively predatory lifestyle, wildcats are expected to be more frequently infected than domestic cats and are likely the source of bridging infections to domestic cats in areas of sympatry.

Cytauxzoonosis caused by *Cytauxzoon felis* was first described as a severe disease in domestic cats in the USA, while its natural host is the bobcat (*Lynx rufus*) [250]. Schizonts are formed in the macrophages of felids after their inoculation with *Cytauxzoon* spp. through a tick bite, resulting in multiple organ failure and clinical cytauxzoonosis in domestic cats [251]. In Europe, however, different parasite species exist and infections in domestic cats are generally less severe, although some heavy and even fatal cases have been reported [252]. Three species have been genetically described in wildcats in Europe (*Cytauxzoon europaeus*, *Cytauxzoon otrantorum*, and *Cytauxzoon banethi* [253]). The clinical importance of these parasites is still unclear, but the high prevalence of *Cytauxzoon* spp. in wildcats contradicts high pathogenicity in this animal species [252]. Recently, *C*. *europaeus* has also been identified in domestic cats in Europe and interestingly, the close genetic relationship between wildcat and domestic cat isolates observed leads to the hypothesis that spillover from the wild reservoirs to domestic cats has occurred [252].

#### 4.1.3. *Troglostrongylus brevior*: A Lungworm of European wildcats Is Gaining a Prominent Place in Domestic Cat Parasitology

The gastropod-transmitted crenosomatid *T*. *brevior* inhabits the bronchi and bronchioles of felines (Felinae). It was first described in an African wildcat (*Felis lybica*, formerly *Felis ocreata*) and a jungle cat (*Felis chaus*, formerly *Catolynx chaus*) in the middle east [254]. In Europe, the parasite is very common in wildcats with recorded prevalences between 14.9% and 71.4% [226,255,256], while it has also been found in Eurasian lynx [256,257]. Due to the widely distributed populations and the high prevalence of infection in wildcats, these animals are considered the reservoir and the natural host of this parasite in Europe [3].

*Troglostrongylus brevior* gained particular scientific attention after 2012 when it was recognised as the agent of important, and occasionally fatal, parasitic disease in domestic cats [258]. The role of wildcats in the epizootiology of troglostrongylosis is critical, evidenced by the fact that the infection in domestic cats is reported mainly in areas of wildcat presence, i.e., south and eastern Europe. Thus, troglostrongylosis is considered a key example of a spillover infection from wildlife to domestic animals [3]. The clinical impact of troglostrongylosis in wildcats has not been clearly demonstrated, as the infection in these animals is often diagnosed post-mortem, or in mixed infection with other cardiopulmonary nematodes that likely worsen the clinical, imaging, gross pathology, and histopathology findings [226,244]. Nevertheless, troglostrongylosis in wildcats may have similar clinical impact as in domestic cats, where the disease has been better studied [259]. Accordingly, in cats *T*. *brevior* shows significant pathogenicity, probably due to its relatively large size and its location (bronchi and bronchioles), resulting in damage to large areas of the lungs [258]. Clinical signs include coughing, tachypnoea, and dyspnoea, abnormal respiratory auscultation sounds, and various non-specific signs, such as poor body condition, anorexia, and lethargy [259]. An important aspect related to the severity of troglostrongylosis is its ability for vertical transmission from queens to kittens [3], which often results in severe and even fatal infections in young cats that are more susceptive to troglostrongylosis than adults. Considering the importance of troglostrongylosis in veterinary medicine, it is essential to keep a vigilant eye on the distribution trends of *T*. *brevior* and include this infection in the differential diagnosis of respiratory disease in domestic cats, especially in the areas of wildcat geographic distribution.

#### 4.1.4. *Angiostrongylus chabaudi*: A Wildcat-Specific Parasite

In contrast to T. brevior, A. chabaudi, a metastrongyloid, gastropod-transmitted nematode that inhabits the pulmonary arteries and the right chambers of the heart of wildcats [260] is characterised as a host-specific parasite. After the first description of its adult stage in a wildcat [261], the parasite came back to light six decades later, with the isolation of a few, immature parasites from two domestic cats in Italy [262,263]. Soon after, a patent infection was diagnosed post-mortem in a wildcat in Greece [260], and subsequently, additional single cases or epizootiological surveys were reported in Romania, Bulgaria, Bosnia and Herzegovina, Italy, and Greece [226,264,265,266,267], confirming that wildcats are the natural host of this parasite, with a prevalence that reaches up to 56.5% [226].

Evidence of the pathogenesis and clinical impact of angiostrongylosis in wildcats exists thanks to the results of post-mortem findings where *A*. *chabaudi* was present in a monospecific parasitosis of the cardiopulmonary system [260,265]. According to those reports, the lungs may be swollen and heavy with a cobblestone appearance. Significant hypertrophy and thickening of the arterial wall were ascribed to pulmonary hypertension caused by the presence of nematodes in the pulmonary arteries. Furthermore, the presence of eggs and migrating larvae was associated with a marked inflammation in the lung parenchyma, with coalescing granulomatous areas, alveolar collapse or emphysemas, and parenchymal haemorrhages [260,265]. Heavy lung lesions were also found in other cases of wildcat angiostrongylosis; however, mixed infection with other cardiopulmonary nematodes did not allow a clear picture of the specific impact of *A*. *chabaudi* [226,264]. Similarly, severe clinical disease and imaging findings have been described in a case of a wildcat hospitalisation, but the multiparasitism diagnosed in this animal renders the description of clinical angiostrongylosis impossible [244].

The gastropod-intermediate hosts of *A*. *chabaudi* are widespread [268,269] and at least some also transmit metastrongyloids that infect both wildcats and domestic cats, i.e., the common cat lungworm *Aelurostrongylus abstrusus* and *T*. *brevior*. However, despite the fact that all three parasites (i) have similar life cycles, (ii) common intermediate hosts, and (iii) circulate in areas where wildcats and domestic cats live in sympatry, to date, *A*. *chabaudi* is not incriminated for spillover from wildcats to domestic cats, and except the two cases in Italy [262,263], no other incidence of infection in domestic cats has ever been reported. It is important to note that lack of infection in cats has been practically confirmed by a great number of epizootiological surveys on cat parasites conducted in recent years in Europe, and especially by surveys targeting specifically the detection of *Angiostrongylus* spp. in domestic cats in areas where infected wildcats have been found [3,270,271]. However, in an environmentally and parasitologically changing world, it cannot be ruled out that eventually spillover infections of *A*. *chabaudi* from wildcats to domestic cats will occur [270]. Bridging infections between wildlife and domestic animals have been recently documented in the case of *A. vasorum* between foxes and dogs and of *T*. *brevior* between wildcats and domestic cats [3,270]. It is important to stress that parasites often cause a milder disease in their natural than in non-natural hosts, as has been evidenced by *A. vasorum* and *C*. *felis*, where the natural hosts, foxes and bobcats, respectively, develop a chronic, subclinical parasitosis while dogs and domestic cats, respectively, often suffer from a severe disease [250,270]. Accordingly, angiostrongylosis may appear more pathogenic in domestic cats as non-natural hosts of *A*. *chabaudi*. For all the above reasons, the scientific community and veterinary practitioners need to be alert for accurate and timely diagnosis of the infection in domestic cats, especially in the areas of Europe where the infection has been documented in wildcats.

#### 4.1.5. *Cylicospirura* spp.: Could It Be a Concern for Domestic Cats?

The parasites of the genus Cylicospirura are spirurid nematodes that live in a ramified burrow inside nodules, formed in the course of this infection in the gastric wall of felids. The described species of the genus so far are *Cylicospirura petrowi*, *Cylicospirura felineus*, *Cylicospirura subaequalis*, *Cylicospirura heydorni*, *Cylicospirura pardalis*, and *Cylicospirura advena*, isolated from a number of different felid species, e.g., *Panthera pardus*, *Panthera tigris tigris*, *L*. *rufus*, *Felis*. *concolor*, and *Felis catus* in practically all continents [272]. In Europe, *Cylicospirura* spp. has been reported in lynx from Germany [273], in wildcats from Italy, Germany, Bulgaria, and Greece [223,225,226,274], and in a domestic cat in Italy [275].

The parasite’s life cycle is unknown, but from the analogy of other Spirocercidae it has been suggested that various insects and small vertebrates may serve as intermediate and paratenic hosts, respectively. Cylicospirurosis seems to remain usually subclinical; however, in some rare cases described in domestic cats, the infection is associated with chronic vomiting [275]. The lesions are restricted to the gastric wall where single or multiple nodules, with a central or subcentral mucosal pore, are formed [226]. Histologically, the nodules are characterised by dense sclerotic collagen with anastomosing bands and inflammatory infiltration around the parasites [275,276].

Based on case reports, it is clear that the parasite may infect domestic cats, albeit quite rarely [276]. For this reason, cylicospirurosis may be encountered as a bridging infection from wildlife to domestic animals more frequently in the future. Particularly in areas where the infection in wildcats has been documented in high prevalence, as in Greece (34.8%) [226], domestic cats may be under high infection pressure due to the environmental and climatic changes that bring wildlife closer to human settlements.

#### 4.1.6. Other Parasites

Wildcats host a number of other parasites for which they may play a minor or no exceptional role when compared with other wild or domestic animals. Nevertheless, due to their lifestyle, wildcats are usually infected with a higher frequency with parasites that may infect equally successfully domestic cats. For example, it has been evidenced that wildcats may serve as definitive hosts of *E. multilocularis* [277], but Felidae seem less competent hosts than Canidae in this important zoonotic cestode [231], so they likely play a minor role in endemic areas. In this context, only immature worms were detected in 5% of the wildcats examined in Germany [223].

As rodents are the main prey of wildcats, parasites such as Taenia taeniaformis are very common in wildcats, showing a prevalence of up to 73.9% [226]. These animals are the main reservoir of this parasite, and although it does not have any zoonotic implications, it may cause clinical disease in felids via intestinal trauma and obstruction [226].

Among others, wildcats may also be important reservoirs for *T. callipaeda* [79,226] and for *C*. *aerophila*, the latter reaching prevalences of up to 33.8% of animals on the basis of post-mortem examinations [226].

## 5. Mustelidae

The family Mustelidae is comprised of small- to medium-sized animals, some of which are located in specific environments (e.g., water) and others are adapted to most habitat types, including urban settings [278]. This family is the largest and the most diverse of the Carnivora order. Mustelids are opportunistic predators feeding on various small mammals, fish, amphibians, birds, fruits, plants, or mushrooms [279]. They are well-known reservoirs of important pathogens for animals and humans as they harbour a wide diversity of viruses, protozoa, and helminths, playing an important role in spreading diseases. Although mustelids have a wide distribution in Europe and are in close contact with domestic animals, parasitological studies of these animals are limited and most of the time include incidental findings. In the last decade, some zoonotic and emerging diseases were detected for the first time in mustelid species [280,281], including the invasive species *Neogale vison* [282]. The absence of relevant studies in some countries could be related to a low research interest in this animal family or to the legally protected status of many mustelids.

### 5.1. The Eurasian Badger (Meles meles)

The Eurasian badger (from now on “badger”) is the biggest mustelid species in Europe with a wide distribution and stable population, classified as “least concerned” according to the IUCN (see File S1) [283]. Badger distribution and population density are correlated with their food resources, which mainly consist of cereals, earthworms, insects, and even rabbits depending on the local conditions [284]. Even though a negative correlation between human population density and badger density was reported [283], in some European countries the anthropogenic land-use change increased food resources for this animal species because of agriculture [285]. Badgers are mainly nocturnal animals that dig large burrows used as temporary shelter by many other animal species, such as foxes, other smaller mustelid species, or even lynx [284,286,287].

#### 5.1.1. *Giardia* spp. and *Leishmania infantum*: Still a Lot to Discover Here

There are only a few published studies investigating *Giardia* spp. in badgers. *Giardia duodenalis*, was first reported in badgers in the UK in 2010 [288] in a group of cubs that was presented to a wildlife center. In that paper, the authors describe severe digestive illness, manifested by acute diarrhea and death among the badgers [288]. It is not clear if the clinical manifestations were related to giardiosis or to other pathogens. Although giardiosis could have clinical implications in badgers, *G*. *duodenalis* assemblage E identified in these cubs does not represent a zoonotic risk [288]. However, recently, infection by zoonotic *G*. *duodenalis* genotypes (assemblages A and B) was identified in 48.8% of Eurasian badgers from Italy [289], highlighting the role of badgers in the epidemiology and spreading of zoonotic genotypes. *Giardia* spp. was molecularly detected in a single animal in Poland, but none of the badgers examined in Spain were positive [290,291]. Even though badgers use latrines for defecation, precipitation can spread the parasite’s cysts into the surroundings, contaminating vegetables, fruits, and water, thus reaching other animal hosts and humans [289].

*Leishmania infantum* in Eurasian badgers has been investigated in Spain and Italy where the parasite was detected by PCR in 26% and 53.33% of the animals, respectively [53,292]. Additional studies were performed in different regions of Spain, but all with negative results [293,294,295]. It is clear that the infection is present in badgers, but it remains to be concluded if these animals represent reservoir hosts and can infect sandflies or not.

#### 5.1.2. *Cryptosporidium* spp. and *Toxoplasma gondii*: Eurasian badgers Are Frequent Hosts

Cryptosporidiosis and giardiosis are two of the most prevalent parasitic diseases in both animals and humans, worldwide [296]. The genus *Cryptosporidium* includes approximately 30 species with more than 70 genotypes. Wild mammals can be infected with a wide range of species while humans are usually infected with *C*. *hominis* and *C*. *parvum* [291]. The typical route of infection is via water or food contaminated with oocysts [297]. The clinical presentation of the infection may vary from asymptomatic to acute or chronic digestive distress [298]. Although *Cryptosporidium* is an important and widespread parasite, limited data are available regarding its prevalence and distribution in wild mesocarnivores, especially in Mustelidae.

*Cryptosporidium* infection in badgers was detected for the first time in 15% of the animals examined in Great Britain, where *C*. *parvum* was morphometrically identified by copromicroscopy [299]. More recently, 2.9% of badgers examined by PCR in Spain harboured *Cryptosporidium* spp., including the zoonotic *C*. *parvum* and *C*. *hominis* [291]. Furthermore, 23.2% of the examined badgers in Italy tested positive for *Cryptosporidium* copro-antigen [289]. These results suggest that badgers could represent an important source of infection in their natural habitats as well as in urban and peri-urban areas. Molecular identification of *Crypstoporidium* spp. in positive badgers is essential in order to establish the actual zoonotic significance of the infection in this animal species.

*Toxoplasma gondii* is a well-studied parasite due to its effects on human health. Among all badger protists it is the most studied one, with high reported prevalences [235,300,301,302,303,304,305]. Between 2000 and 2010, seroprevalence over 70% was reported in badgers from the UK [300] and Spain [235,301]. In more recent studies in Portugal and Poland, 50% and 37.5% of the examined animals, respectively, were seropositive [302,306]. The infection in badgers has also been confirmed molecularly in the UK, Poland, Slovakia, and the European part of Turkey [303,304,305,306,307]. Although none of the badgers showed any clinical symptoms related to toxoplasmosis, some authors consider that anorexic and lethargic badgers or those showing an atypical behaviour should be tested for toxoplasmosis [300].

A survey in Poland confirmed that *T. gondii* prevalence in mustelids was significantly higher when compared to canids, rodents, and insectivores, which indicates that animals of the Mustelidae family are important reservoirs for *T. gondii* [305].

#### 5.1.3. *Trichinella* spp., *Angiostrongylus vasorum*, *Dirofilaria immitis*, *Thelazia callipaeda*: Nematodes of Veterinary and Medical Importance can Infect Eurasian badgers

In some countries (e.g., Romania, Czech Republic, Croatia, Russia) badgers are tradi-tionally hunted for meat consumption or for their fat, used as a food supplement, thus in-fection by *Trichinella* spp. must be considered [308]. According to the International *Trichinella* Reference Centre (ITRC) [309], the species *T*. *britovi*, *T*. *nativa*, and *T*. *pseudospiralis* and 20 *Trichinella* isolates have been found in badgers. For example, *T. britovi* was identified in badgers from Latvia, Italy, and Romania, *T. britovi* and *T*. *nativa* in Estonia, and *T*. *nativa* and *T. spiralis* in Ukraine [310,311].

In a study performed between 2007 and 2014 in wild animals in Estonia, a large proportion (60%) of the badgers examined were found to be infected with *Trichinella* spp. This was a significantly higher prevalence than recordings taken between 1965 and 2000, which was 6.7% [312]. The infection prevalence in Latvia of 100% indicates that badgers could be more commonly infected than previously assumed and favour the transmission of *Trichinella* [313].

Most importantly, the risk of human infection may be considered high as badger meat is consumed in several European countries and no routine inspections are implemented as badger is not an official meat source for the population. Infection in humans due to consumption of badger meat has not been reported yet in European countries, probably due to culinary habits (eating thermally processed meat); however, such human infections have been recorded in other continents [314,315]. In Europe, one of the main sources of human infection is wild boar meat [7]. In their diet, wild boars include 5–88% animal matter, mainly small mammals, but larger mammals are sometimes also ingested as carrions [316,317,318]. There are no confirmed records of mustelid consumption by wild boar, but this is something that cannot be ruled out and thus it can be hypothesised that mustelids may play a role in *Trichinella* transmission to wild boar.

The canine cardiopulmonary nematode *A*. *vasorum*, was documented in badgers in Italy, Spain, and Norway by necropsy [319,320,321,322,323,324]. *Angiostrongylus vasorum* is a common parasite of red foxes, while badgers are typically infected with *Angiostrongylus daskalovi*, a different but morphologically similar species [324]. Thus, misidentifications should be taken into consideration and molecular confirmation of the identity should be considered. The role of mustelids in the transmission of *A. vasorum* and their role in the biology of canine angiostrongylosis has not yet been determined.

Although the occurrence of *D. immitis* microfilaraemia was documented in a badger by microscopical examination and molecular detection, which would normally signify a potential reservoir host, this isolated finding could be considered accidental and further research is needed (e.g., prevalence of infection, microfilaraemia duration) in order to establish the exact role of badgers in the transmission and maintenance of dirofilariosis in a given area [281].

Badgers are mostly nocturnal animals, thus not regularly exposed to the fruit fly vector of the eye worm *T. callipaeda* [79]. Nevertheless, the parasite was found in 1.8% of the examined badgers in Romania, confirming this animal species as a suitable host [280]. However, given the rarity of *T. callipaeda* in mustelids evidenced so far, the infection can be considered opportunistic in these animals in endemic areas.

### 5.2. Martens (Martes foina, Martes martes)

The genus *Martes* includes eight species of which only two, namely the stone or beech marten (*Martes foina*) and the pine marten (*Martes martes*), are present in Europe. Both species have stable populations [324] are adapted to various habitats, from coniferous to Mediterranean forests, and are well-adapted to human settlements, including urban and suburban areas of villages and even towns [325].

#### 5.2.1. *Beech marten* (*Martes foina*)

The beech marten, also known as the stone marten, house marten, or white-breasted marten, has spread to south, central, and eastern Europe (see File S1), and prefers open areas. The species is common in urban areas, inhabiting attics, barns, garages, and other human-made enclosures [326].

##### *Giardia* spp., *Leishmania infantum*, *Toxoplasma gondii*: Badgers’ Proximity to Domestic Animals and Humans Is a Looming Risk

*Giardia* spp. infection in beech martens was investigated in Poland [290] with negative results and in Spain with 13% prevalence in PCR testing [291]. Considering the closeness of this animal species to human settlements, the risk of transmission to domestic animals and humans should be considered.

Several studies investigated the infection of *L*. *infantum* in stone martens in the endemic countries of Spain and Greece [292,293,294,295,327,328]. All studies performed in Spain were based on molecular detection of *L*. *infantum* DNA and revealed prevalences of up to 100%, but the only marten tested from Zakinthos island in Greece was negative [327]. However, the report from Greece is quite old and the method of diagnosis is not mentioned, thus a false negative result should be considered.

Muñoz-Madrid et al. [328] were the first to investigate mustelids as potential reservoir hosts of *Leishmania* in Spain, using a qPCR method from hair. Surprisingly, all three examined beech martens were found to be positive. Another study performed on 21 martens from Basque Country in northern Spain identified the infection with *Leishmania* in 6 animals (29%) [292]. More recently, in 2018 a study from the regions Murcia, Valencia, and Andalucia detected a 30% prevalence in 10 examined animals. In northwestern Spain, two out of the three examined beech martens were positive by PCR testing [294]. In four animals investigated in the Catalonia region by qPCR testing, a prevalence of 50% was found in DNA extracted from the liver and spleen [295].

These results, together with the fact that M. foina is commonly spotted in human settlements, could signify an important role of this animal in the epidemiology of L. infantum. However, it is not possible to draw any definite conclusion because there is no evidence of stone martens’ capacity to infect sandflies, thus further research on this matter would be elucidating.

*Toxoplasma gondii* infection in *M*. *foina* was detected in 18% of the tested animals in the Czech Republic by xenodiagnosis [329] and at a prevalence of 4.92% by PCR testing several years later [330]. In Spain, 85% of the tested beech martens were seropositive [235]. Furthermore, 57.1%, 66.7%, and 52.9% of the examined beech martens in Slovakia, Portugal, and Lithuania, respectively, were positive by PCR testing and serology, respectively, [236,304,331]. The high prevalence of *T. gondii* infection is likely related to beech martens’ main prey in winter, i.e., rodents [332]. Due to their proximity to peri-urban and urban areas, these animals can be an important source of infection for domestic cats or even urban rodents.

##### *Thelazia callipaeda*, *Capillaria aerophila* and *Trichinella* spp.: Beech Martens Are Additional Hosts of These Important Parasites

Studies on *T. callipaeda* in the abundant group of mustelids are still limited with scarce information on their role as reservoir hosts for this parasite [280,333]. In Europe, infection with *T. callipaeda* in beech martens was documented in Italy (13.60%) [79], Portugal (one case report) [334], and Romania (7.69%) [280]. The idea that wild animals act as reservoir hosts for *T. callipaeda* has been previously discussed [29]. Considering that beech martens are abundant, they may play an important role in maintaining and spreading the infection to domestic animals and humans [29].

*Capillaria aerophila* is a zoonotic lungworm that was identified in beech marten carcasses in Europe [310,335]. The contamination of the environment with high numbers of infective eggs raises the chances of transmission to dogs, cats, and even humans.

*Trichinella spiralis* and *T. britovi* were isolated from beech martens, which could represent a source of infection for other sylvatic animal species, but less so for humans as consumption of beech marten meat is not reported in European countries. Reported prevalences range between 4.8% and 50% for *T. britovi* in Italy [336], Slovakia [337], Serbia [338], and Romania [339]. *Trichinella spiralis* was identified in lower Silesia, Poland, in 15.3% of the examined animals [340]. The detection of the “domestic cycle” species *T. spiralis* in mustelids is attributed to the habitat loss and urbanisation of these wild animals. The transmission to domestic animals can be promoted by the presence of backyard pigs with free outdoor access [339].

#### 5.2.2. Pine Marten (*Martes martes*)

The pine marten prefers woodlands with dense vegetation. It is found mainly in northeastern and central Europe, but some populations are present in parts of the Mediterranean (see File S1). Their presence was reported in some European cities. Their food is mainly based on small mammals, such as voles, mice, squirrels, birds, and amphibians [341]. Due to their feeding habits, they are very likely to be infected with food-borne parasites such as *T. gondii*. Several studies investigated the infection of European *M*. *martes* with *T. gondii* with moderate to high detected prevalences (17% to 100%) [235,304,305,306,329,330,331].

Similar to the other animal species of this genus, pine martens are important reservoirs for *Trichinella* in sylvatic and urban environments. In this animal species, trichinellosis was identified in a few European countries with varying prevalence (8.3–91.8%) [191,310,311,313,336,337,339,342,343,344,345,346,347,348,349,350]. The species *T. britovi* was mainly identified, while in two cases *T. spiralis* was detected and there is also a single report of T. nativa in a pine marten from Latvia, coinfected with *T. britovi* [313]. The presence of *T. spiralis* suggests an involvement of pine martens in the domestic cycle of the parasite and a possible important role of these animals in the epidemiology of infection. On the other hand, according to the reports, *T. spiralis* is scant, which could be related to a low susceptibility of martens in maintaining the infection [310,338]. Further large-scale studies are needed in order to establish the exact nature and role of mustelids in the life cycle of *Trichinella*.

### 5.3. The Genus Mustela: Abundant and Parasite Carier

The species of the genus Mustela are globally distributed, and their number continuously changes, now numbering more than 15 [324]. Among them, there are very abundant and population-stable species, such as the European polecat (*Mustela putorius*), or highly endangered species such as the European mink (*Mustela lutreola*) [351]. Food-borne parasitic infections are often detected in these small and fit animals that feed on rodents, lagomorphs, and other vertebrate and invertebrate hosts [352]. As expected, they are important reservoirs for *T. gondii*. The majority of studies investigated the infection in polecats, a species that inhabits many European countries and is known to live near farms or other human settlements and revealed infection rates of up to 100% in the UK [303], Spain [235], Portugal [236], Poland [305], and Slovakia [304]. Furthermore, *T. gondii* was detected in the brain of 25% of weasels (*Mustela nivalis*) from the Czech Republic [329].

*Trichinella britovi* and *T. spiralis* were identified in the genus *Mustela* only in eastern Europe with low prevalence rates [337,353,354]. Small mustelids can be important reservoirs as they represent a possible source of food for wild boar. However, as previously mentioned, it is hard to verify the exact origin of hair and bones detected in the stomach of wild animals, thus more studies are needed in order to confirm the role of mustelids in the transmission and maintenance of trichinelosis.

Finally, infection with *A. vasorum* was reported in a polecat from Denmark [335] and in one ermine (*Mustela erminea*) from the UK [355], suggesting that these animals could play a role in the maintenance of this parasite in an area.

### 5.4. Eurasian otter (Lutra lutra): Their Water Affiliation May Be Decisive for Its Epidemiological Impact

The Eurasian otter (from now on “otter”) is a semiaquatic mammal present in most parts of Europe. Thanks to conservation efforts, declining otter populations are now in a process of recovery (see File S1) [356]. Otters live in a wide variety of aquatic habitats and although they are elusive, they can be found close to human settlements [357].

The affiliation of otters with water renders them a potentially important animal for the dissemination of parasites such as *Giardia* spp. and *Cryptosporidium*, for which water (drinking, recreational) is important source of infection. *Giardia* spp. was found in 1 out of 33 otters in Denmark [358] while both *Giardia* and *Cryptosporidium* were found 3.9% and 6.8% of the otters examined in northwest Spain, respectively [359], but no information about the zoonotic potential of these infections is available as no genetic characterisation of the parasites was performed.

As expected, otters are also infected with *T. gondii* through their prey and also because the parasite oocysts are transferred from the soil and are accumulated in the aquatic habitats by precipitation and agricultural water runoff [360]. In this sense, otters can be utilised as sentinels for *T. gondii* load in a given area [361]. In fact, *Toxoplasma* seropositivity in otters has been documented from 25.5% to 100% depending, among others factors, on the sample size [236,360,361], indicating that otters play an important role in the maintenance and circulation of the parasite.

The otter has occasionally been found as infected with *D. immitis* [168,362,363]. However, its factual reservoir role is unknown, as microfilaraemia was documented in only one case [168,362,363]. Further investigations are needed to clarify the role of otters as a source of infection for dogs and other hosts in the same area.

### 5.5. The American Mink (Neogale vison): An Invasive Species Claims Its Role in Parasite Epidemiology in Europe

The American mink is native to North America, and it was introduced in Europe as a fur animal. Due to the fact that some animals escaped or were intentionally released in nature, the species has now a stable population in some parts of Europe (see File S1) [364]. The American mink is distributed mostly along streams and lakes, but it is also often detected in swamps and marshes. It is a strictly carnivorous species that feeds on fish, amphibians, crustaceans, and some small mammals [365,366], and it is an important reservoir host for many zoonotic pathogens, including parasites.

American minks were found serologically or molecularly positive for *T. gondii* in the UK, Poland, and Denmark, reaching a prevalence of up to 78.8% in Spain [17,303,306,367,368,369]. Infection with *Cryptosporidium* was detected in animals from Ireland, Spain, and the Czech Republic with prevalences of 6.17%, 24.20%, and 6%, respectively. Taking into consideration that both *T. gondii* and *Cryptosporidium* are often water transmitted and that American minks prefer habitats close to water, it may be speculated that these animals are important spreaders of the parasites.

The zoonotic parasite *L*. *infantum* was detected by PCR testing in DNA extracted from the liver and spleen of one mink from Spain [295], suggesting that these animals may represent an additional reservoir host species in endemic areas.

Finally, the nematode of emerging veterinary concern, *A*. *vasorum*, was found in 1.16% of the American minks examined in Denmark [335], the zoonotic *C. aerophila* in 11–15% of the animals in Lithuania [370,371], and the important food-borne nematode *Trichinella* spp. in Ukraine and Poland in 3.3% and 25 of the animals, respectively [282,310,340].

## 6. Conclusions

According to the expanding relative literature, mesocarnivores hold an important and in some cases critical role as reservoirs and bridge hosts of important emerging parasites in Europe. These animals host parasites that may be introduced into the areas where they expand or in which they invade and can be transmitted to sympatric domestic animals. An important portion of these parasites have a proven or potential zoonotic importance and thus should be taken into consideration in human medicine and the One Health approach to public health, especially in the context of changing conditions due to climate change, land-use change, natural environment degradation, and rewilding of urban areas.

Continuing investigations and monitoring are warranted and necessary in order to elucidate further the occurrence, prevalence, and epidemiology of the parasites under consideration and to design strategies that would control and prevent the occurrence of important zoonoses and emerging bridging infections between wildlife, domestic animals, and humans.

## Figures and Tables

**Table 1:** The parasites discussed and reported in this article for each mesocarnivore species in Europe.

Parasites	Animal Species										
	*Vulpes vulpes*	*Canis aureus*	*Nyctereutes procyonoides*	*Procyon lotor*	*Felis silvestris*	*Meles meles*	*Martes foina*	*Martes martes*	*Mustela* spp.	*Lutra lutra*	*Neogale* *vison*
*Gia*	●					✓	✓			●	
*Lei*	✓	●		●		✓	✓				●
*Bab*	✓	●									
*Hep*	✓	●			✓						
*Txp*	✓			●	✓	✓	✓	●	●	●	
*Cry*	●					✓				●	●
*Cyt*					✓						
*Ala*	●	●	●								
*Ech*	✓	●	✓		●						
*Tae*	●	●			●						
*Mes*	●	●									
*Ang*	✓	●			✓	✓			●		●
*Dir*	✓	✓		●		✓				●	
*Tri*	✓	●	✓	●		✓	✓	●	●		
*Cap*	✓	●			●		✓				●
*The*	✓	●			●	✓	✓				
*Spi*	●	●									
*Tro*					✓						
*Cyl*					✓						
*Bay*				✓							
*Str*				✓							

*Gia*: *Giardia* spp., *Lei*: *Leishmania* spp., *Bab*: *Babesia* spp., *Hep*: *Hepatozoon* spp., *Txp*: *Toxoplasma gondii*, *Cry*: *Cryptosporidium* spp., *Cyt*: *Cytauxzoon* spp., *Ala*: *Alaria alata*, *Ech*: *Echinococcus* spp., *Tae*: *Taenia* spp., *Mes*: *Mesocestoides* spp., *Ang*: *Angiostrongylus* spp., *Dir*: *Dirofilaria* spp., *Tri*: *Trichinella* spp., *Cap*: *Capillaria* spp., *The*: *Thelazia callipaeda*, *Spi*: *Spirocerca* spp., *Tro*: *Troglostrongylus brevior*, *Cyl*: *Cylicospirura* spp., *Bay*: *Baylisascaris procyonis*, *Str*: *Strongyloides* spp., ✓ the parasite is discussed. **●** the parasite is reported.

## Data Availability

Not applicable.

## Supplementary Materials

The following supporting information can be downloaded at: https://www.mdpi.com/article/10.3390/pathogens12020178/s1, File S1: Characteristics and geographical distribution of mesocarnivores in Europe.
