# Supplementary material for: Wild Mesocarnivores as Reservoirs of Endoparasites Causing Important Zoonoses and Emerging Bridging Infections across Europe
Source: Pathogens. 2023 Jan 23;12(2):178. doi: 10.3390/pathogens12020178 (PMC9964259; doi:10.3390/pathogens12020178)
Supplement: Supplementary file 1 [file pathogens-12-00178-s001.zip › pathogens-2164782-supplementary.pdf]

Family Canidae

	RED FOX	GOLDEN JACKAL	RACCOON DOG
	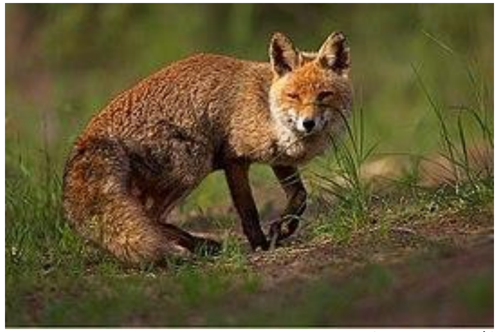 CC Martin Mecnarowski	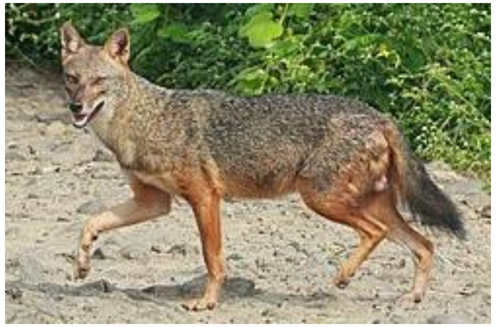 CC Shino Jacob Koottanad	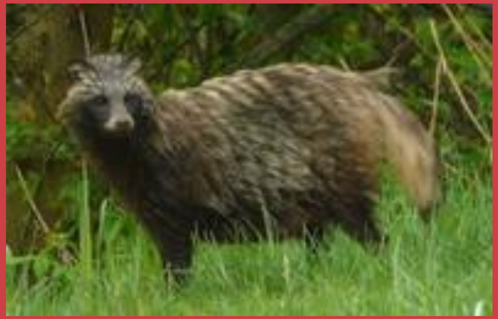 CC ShootGun180
**Binomial name**	*Vulpes vulpes* Linnaeus, 1758	*Canis aureus* Linnaeus, 1758	*Nyctreutes procyonoides* Gray, 1834
**Diet**	Omnivore	Omnivore	Omnivore
**IUCN status**	LC	LC	ALLOCHTHONOUS IN EUROPE
**Litter size**	3 - 12	1 - 8	4 - 9
**Body weight**	3.4 kg - 8.7 kg	6.5 kg - 9.8 kg	2.9 kg - 12.5 kg
**Distribution range**	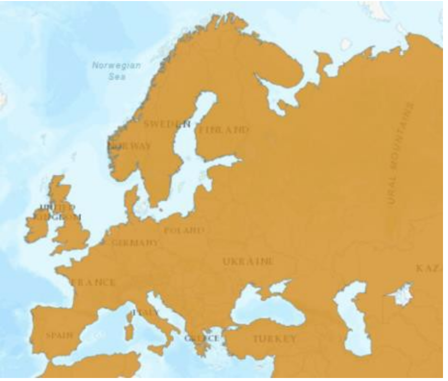	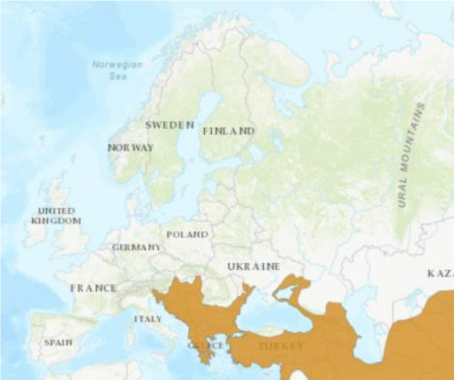	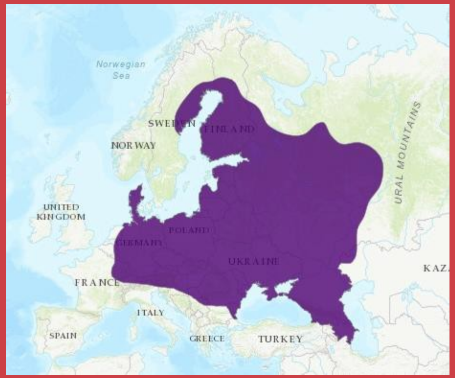

RESIDENT INTRODUCED

LC: *least concern*

EUROPEAN WILDCAT

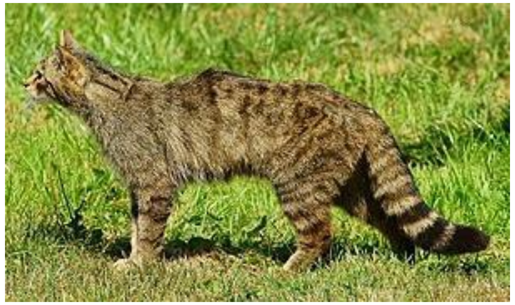

CC Peter Trimming

Family Felidae

Binomial name	*Felis silvestris* Schreber, 1777
Diet	Carnivore
IUCN status	LC
Litter size	2 – 4
Body weight	2 kg - 7.7 kg
Distribution range	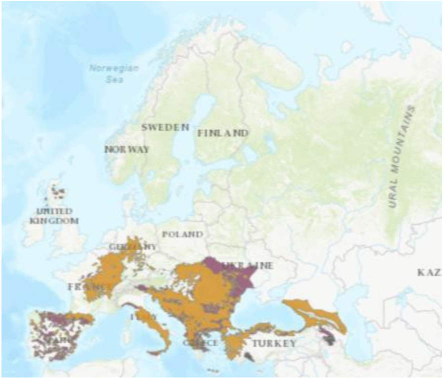

RESIDENT POSSIBLY RESIDENT LC: *least concern*

RACCOON

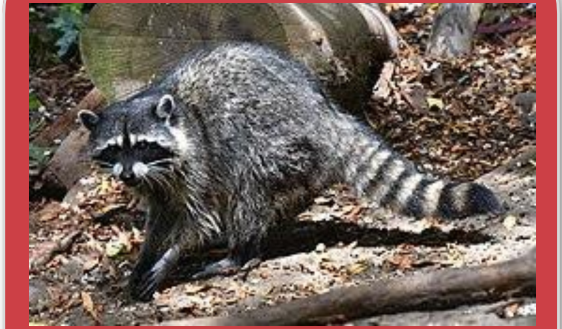

CC Don Loarie

Family Procyonidae

Binomial name	*Procyon lotor* Linnaeus, 1758
Diet	Omnivore
IUCN status	ALLOCHTHONOUS IN EUROPE
Litter size	1 – 7
Body weight	2 kg - 12 kg
Distribution range	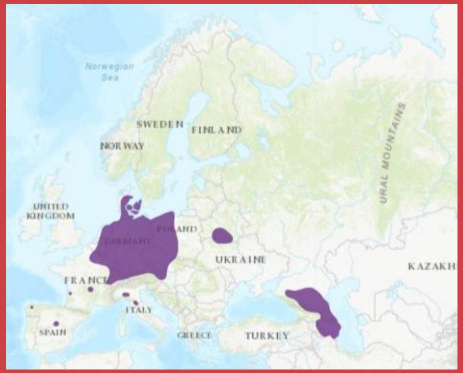

INTRODUCED

Family Mustelidae	EUROPEAN BADGER	BEECH MARTEN	PINE MARTEN	EURASIAN OTTER	AMERICAN MINK
	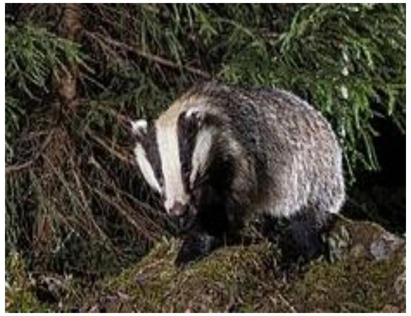 CC Trond Sætre Stegarud	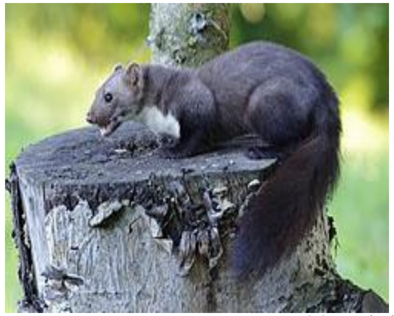 CC M.zlinko	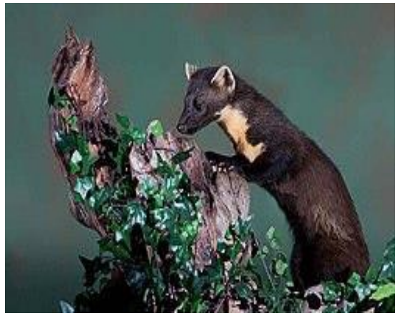 CC Ellis Lawrence	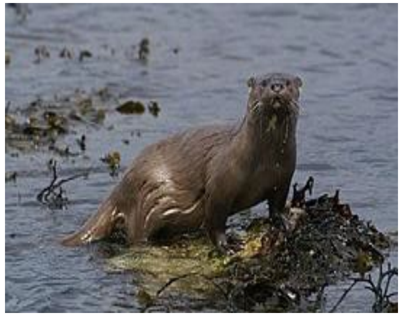 CC Mike Pennington	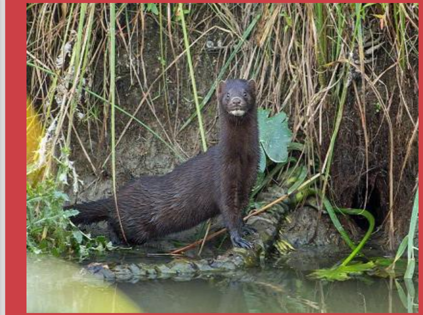 CC Christian Fisher
**Binomial name**	*Meles meles* Linnaeus, 1758	*Martes foina* Erxleben, 1777	*Martes martes* Linnaeus, 1758	*Lutra lutra* Linnaeus, 1758	*Neogale vison* Schreber, 1777
**Diet**	Omnivore	Omnivore	Omnivore	Carnivore	Carnivore
**IUCN status**	LC	LC	LC	NT	ALLOCHTHONOUS IN EUROPE
**Litter size**	2 - 6	3 - 8	2 - 8	4 - 9	2 - 10
**Body weight**	10 kg - 16 kg	1.1 kg – 2.3 kg	0.8 kg - 1.8 kg	5 kg - 14 kg	0.7 kg - 1 kg
**Distribution range**	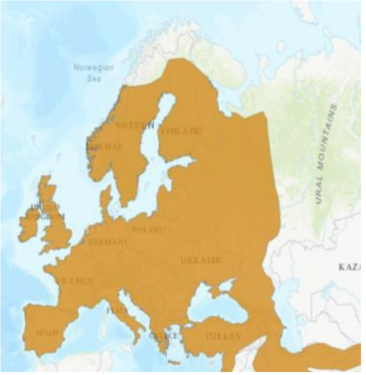	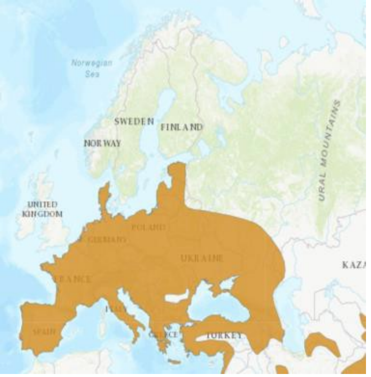	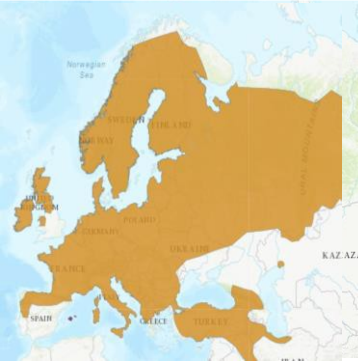	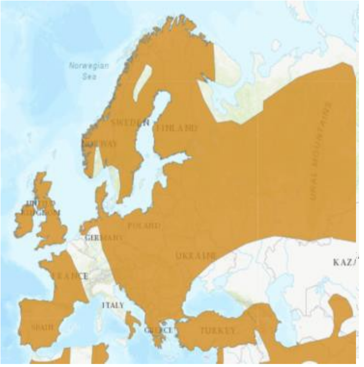	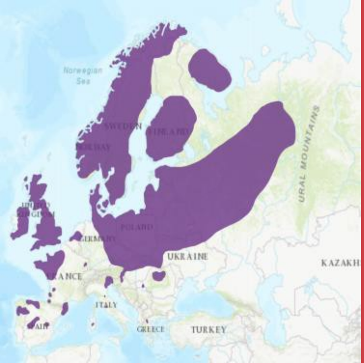

LC: *least concern* N.T: *near threatened*

RESIDENT INTRODUCED
